# Endocrine disruptors, aryl hydrocarbon receptor and cortisol secretion

**DOI:** 10.1007/s40618-024-02371-w

**Published:** 2024-04-18

**Authors:** F. Pecori Giraldi, F. Ferraù, M. Ragonese, S. Cannavò

**Affiliations:** 1 Department of Clinical Sciences and Community Health, University of Milan, Via Commenda 19, Milan, Italy; 2 Department of Human Pathology of Adulthood and Childhood “Gaetano Barresi,”, University of Messina, Messina, Italy

**Keywords:** Endocrine disruptor, Aryl hydrocarbon receptor, Adrenal cortex, Cortisol

## Abstract

**Purpose:**

Endocrine disruptors exert a plethora of effects in endocrine tissues, from altered function to carcinogenesis. Given its lipophilic nature, the adrenal cortex represents an ideal target for endocrine disruptors and thus, possibly, xenobiotic-induced adrenocortical dysfunction. However, there is no clear understanding of the effect of endocrine disruptors on adrenal steroidogenesis, in particular as regards the aryl hydrocarbon receptor (AHR) pathway, one of the key mediators.

**Methods:**

The present review recapitulates available evidence on the effects of AHR ligands on adrenal steroidogenesis, with focus on cortisol secretion.

**Results:**

Short-term exposure to AHR ligands most often induced a stress-like corticosteroid response followed by decreased responsiveness to stressors with long-term exposure. This was observed in several experimental models across species as well as in animals and humans in real-life settings. Prenatal exposure led to different effects according to sex of the offspring, as observed in murine models and in children from mothers in several countries. In vitro findings proved highly dependent on the experimental setting, with reduced cortisol response and steroidogenic enzyme synthesis mostly observed in fish and increased cortisol synthesis and secretion observed in murine and human adrenal cell lines. Of note, no AHR-binding element was detected in steroidogenic enzyme promoters, suggesting the involvement of additional factors.

**Conclusion:**

Our review provides evidence for the impact of AHR ligands on adrenocortical function and indicates further avenues of research to better clarify its effects.

## Introduction

Endocrine disruptors exert multiple effects on endocrine tissues, with exposure to different agents resulting in different, sometimes even contrasting, consequences. One of the key mechanisms called into play by endocrine disruptors is the aryl hydrocarbon receptor (AHR) pathway, and this review will recapitulate current knowledge on the effect of AHR ligands on adrenocortical function. The adrenal gland represents an ideal target for AHR ligands given its propensity to concentrate lipophilic compounds such as aromatic hydrocarbons. Once retained, AHR ligands can affect steroidogenesis and impact cortisol secretion, which is crucial to both homeostasis and stress responses.

We will assess findings in experimental and real-life settings in both animals and humans to provide a clear view on the impact of AHR ligands on cortisol secretion and its possible influence on health status.

## Methods

We performed an extensive MEDLINE search for the following terms: “aryl hydrocarbon receptor, adrenal, adrenocortical, cortisol, endocrine disruptor, xenobiotic, persistent organic pollutants.” Search terms were linked to medical subject headings (MeSH) where available. Keywords and free words were used simultaneously. Publications were retrieved and additional articles were identified through manual search and study of review articles and cross references. Any discrepancy was resolved by discussion.

## Aryl hydrocarbon receptor and its ligands

The aryl hydrocarbon receptor is a ligand-activated transcription factor, a member of the basic helix-loop-helix superfamily involved in a myriad of biochemical pathways, from energy metabolism to xenobiotic (dis)activation, from cell cycle regulation to immune function. The interest in adrenal pathophysiology stems from its role as an inducer of cytochrome 450 (CYP) enzymes, the main actors of adrenal steroidogenesis.

**Fig. 1:**
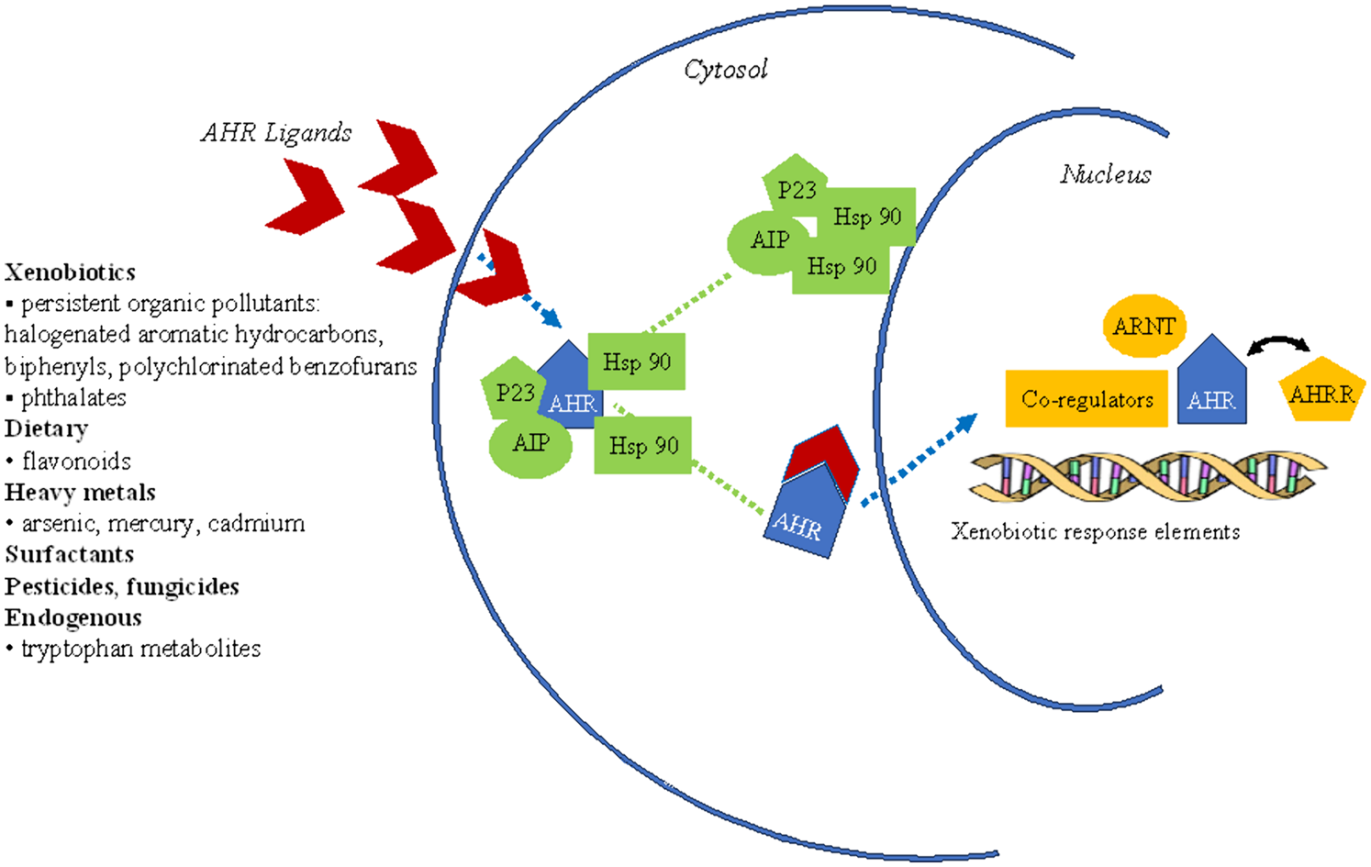
Aryl hydrocarbon receptor (AHR) pathway. *HSP90* heat shock protein 90, *AIP* AHR inhibitory protein, *ARNT* AHR nuclear translocator, *AHRR* AHR repressor

In its resting state, AHR is located in the cytoplasm and complexed to HSP90 and the AhR inhibitory protein (AIP). Upon ligand binding, the AHR–ligand complex translocates to the nucleus, sheds HSP90 and AIP, and binds the AHR nuclear translocator (ARNT). This heterodimer then binds to DNA at specific sequences, i.e., xenobiotic-responsive elements (XRE), recruits coactivators, and initiates gene transcription. AHR also induces expression of the aryl hydrocarbon receptor repressor (AHRR), which competes for ARNT and XRE binding, thus forming an autoregulatory loop (Fig. 1) [1].

AHR was first discovered as the receptor for 2,3,7,8, tetrachlorodibenzo-*p*-dioxin, a halogenated aromatic hydrocarbon. This and other dioxins have multiple chlorine atoms (i.e., polychlorinated)––which confer toxicity––and together with polychlorinated benzofurans and polychorinated biphenyls are called *persistent organic pollutants.* In fact, these compounds are characterized by long-lasting half-life in the environment, in animals and in humans. Thus, although dioxin-containing herbicides have been banned in the 1980s and dioxin emission from hazardous waste incineration is being actively reduced, they still represent a significant health hazard. It is worth recalling that polychlorinated hydrocarbons are lipophilic and accumulate in lipid-rich tissues [2] and that adrenal CYP enzymes themselves are involved in xenobiotic metabolism and bioactivation [3]. Altogether, the adrenal appears a likely target for uptake, storage, and activation of organic pollutants.

In addition to dioxins, a wide variety of AHR ligands have been identified, e.g., chemicals such as polyfluoralkyl acids from household and industrial products and phthalates from the plastic industry, dietary elements such as flavonoids, and even endogenous compounds, such as tryptophan metabolites. In addition, heavy metals such as arsenic [4], mercury [5], and cadmium [4], phenol surfactants [6], the pesticide endosulfan [7], and the fungicide prochloraz [8] (Fig. 1) were shown to exert AHR activity and activate AHR-target genes. It is also worth recalling that ketoconazole––a widely used agent for Cushing’s syndrome––is itself an aromatic hydrocarbon and, in addition to its action on the first and last step of steroidogenesis, interacts with the AHR complex and activates hepatic AHR-dependent gene transcription [9].

Exposure to AHR ligands is extremely difficult to estimate. Trace amounts of the above-mentioned substances are present in water as well as air, and AHR transcriptional activity has been detected in a variety of food products. Further, ligands may sometimes exert both agonist and antagonist activities in a cell-specific manner*.* Altogether, the effect of AHR ligands appears complex and an appraisal of evidence collected across species in different experimental settings is necessary to obtain a clear view of current knowledge.

## Rodents

Rodent studies allow testing with a variety of experimental models, from transgenic mice to exposure during gestation or at specific stages in life (Table 1). These studies provided considerable insights into the mechanisms of AHR activity in the adrenal starting with the effect of dioxin, the premier AHR ligand*.*

**Table 1:** Studies with AHR ligands on corticosteroid secretion in rats in vivo and in vitro

Species	AHR ligand	Experimental model	Findings	Reference
**Studies in adult animals**				
Adult male rat	dioxin	Single dose 50 µg/kg bw by oral gavage	Transiently increased corticosterone followed by long-lasting decrease	[10]
Adult male rat	bisphenol A	0.5 mg/kg bw daily for 3 days	Increased serum corticosterone	[25]
Adult male rat	di-butyl phthalate	100 mg/kg bw daily by oral gavage for 2 weeks	Reduced serum corticosterone, increased *Star*, *Cyp11b2*, *Cyp21a1* expression	[16]
Adult male rat	polybrominated diphenyl ester	100 µg/kg bw by oral dosing for 16 weeks	Increased plasma corticosterone	[19]
Pubertal male rat (5 weeks)	polybrominated diphenyl ester	200 mg/kg bw by oral dosing for 3 weeks	Increased serum corticosterone, increased *Star*, *Cyp11b2*, *Cyp21a1* expression	[18]
Adult female rat	genistein, bisphenol A, resveratrol	3 mmoles/100 g bw daily sc for 10 days; adrenal enucleation on day 5	Reduced plasma corticosterone with genistein; no effect with bisphenol A and resveratrol	[15]
Adult female rat	hexachlorobenzene	200 mg/kg bw by oral dosing for 5 days	Reduced serum corticosterone up to 8 weeks	[17]
Adult female OVX rat	hexachlorobenzene	1 mg/kg bw daily by oral gavage for 4 weeks	Reduced plasma corticosterone	[14]
**Pre- and postnatal exposure**				
Newborn and adult mice	arsenic	0.05 mg/l in water to dams	Blunted corticosterone response to stress in male offspring, normal response in female newborns, decreased basal plasma corticosterone in adult male and females	[20]
Pubertal rat (6 weeks)	bisphenol A	40 µg/kg bw in chow to dams	Increased basal plasma corticosterone in female offspring, blunted corticosterone response to stress in both male and female offspring	[22]
Pubertal male rat (7 weeks)	dichlorodiphenyl trichloroethane (DDT)	20 µg/l in water Prenatal exposure: to dams Postnatal exposure: until puberty	Prenatal: increased plasma corticosterone Postnatal: decreased plasma corticosterone	[26]
Young adult rat (10 weeks)	dioxins polychlorobiphenyls polychlorobenzofurans	Mixture in chow to dams up to 20 days after birth	Abnormal cortisol response to stress	[23]
Young adult rat (10 weeks)	bisphenol A	5 mg/kg bw in chow to dams	Increased basal plasma corticosterone in both male and female offspring	[93]
Young adult rat (13 weeks)	nonylphenol	2 µg/ml in water to dams	Marked increase in serum corticosterone in male offspring, modest increase in female offspring	[21]
**In vitro studies**				
Mouse Y1 cell line	bisphenol A	10 nM – 1 µM for 24 h	Increased basal corticosterone secretion Increased Cyp11a1 protein and activity	[25]
Rat adrenal primary culture	bisphenol A, resveratrol	1 pM – 10 nM for 24 h	Increased corticosterone response to ACTH	[26]

*OVX* ovariectomized, *sc* subcutaneous injection, *bw* body weight, *wk* weeks

### In vivo studies

Administration of single oral dose (50 µg/kg) of tetrachlorodibenzo-p-dioxin (TCDD) to adult rats transiently increased corticosterone followed by long-lasting reduction in both plasma and adrenal levels [10]. The same experimental model yielded impaired conversion to pregnenolone, indicative of reduced Cyp11a1 activity [11] and reduced adrenal 21 hydroxylase activity [12]. In addition to the effect on adrenal steroidogenic enzymes, dioxin and congeners are metabolized within the adrenal cell to highly toxic intermediates, which cause mitochondrial damage and adrenocortical necrosis [13]; in fact, this is the ultimate effect of mitotane treatment in adrenal carcinoma.

Other AHR ligands have also been tested in rodents and the results are summarized in Table 1. Most studies reported a reduction in plasma corticosterone levels upon administration of AHR ligands over several days or weeks [14–17]. In some experiments, the effect appeared to extend over time as basal and ACTH-stimulated corticosterone secretion was blunted up to 2 months after animals had been treated with hexachlorobenzene; of note, adrenal weight was comparable to controls attesting to an effect on secretion, not on cell trophism [17]. In contrast with these findings, treatment with polybrominated diphenyl esters resulted in an increase in plasma corticosterone in both adult and pubertal rats [18, 19].

### Prenatal and early postnatal exposure

Prenatal exposure to AHR ligands revealed sexually dimorphic effects in newborns (Table 1). Male offspring from dams fed arsenic presented reduced corticosterone response to stress, whereas female pups were unaffected [20]. At adrenal level, activation of steroidogenesis, i.e., increased adrenal Star protein and 11ßhydroxylase activity, was observed in male offspring only [21]. Gender differences were less evident in older offspring from dams exposed to AHR ligands during gestation: In fact, increased basal plasma corticosterone but blunted corticosterone response to stress were observed in pubertal rats (6–7 weeks of age) of either sex [22, 23]. Likewise, abnormal cortisol responses to stress were observed in young adult rats of both sexes from dams fed a variety of AHR agonists (Table 1).

Early postnatal exposure was also studied, albeit in male offspring only: Pups nurtured by AHR-agonist fed mothers presented reduced corticosterone response to stress [23, 24]; whether this applies also to female offspring remains to be established.

### In vitro studies

The majority of in vitro studies with AHR ligands reported increased adrenal steroidogenesis (Table 1). Increased corticosterone secretion, both at baseline and with ACTH stimulation, as well as increased Cyp11a1 protein and activity, was observed in the mouse Y1 cell line [25] and rat adrenal primary cultures [26]. Remarkably, increased corticosterone secretion was observed both with bisphenol A and resveratrol [26], an AHR agonist and antagonist, respectively [27], indicating that this distinction carries little weight in the adrenal. Also of interest, upregulation of adrenal steroidogenic enzymes was observed in two ex vivo models with opposite effects in vivo: Adrenal *Star*, *Cyp11b2,* and *Cyp21a1* expression was increased in rats treated with di-butyl phthalate [16] or a bromo diphenyl ester [18], but reduced serum corticosterone levels were observed in the former and increased levels in the latter model (Table 1).

### Transgenic experiments

Transgenic models were also used to assess the involvement of the AHR pathway in adrenal function and development. Both *Ahr* and *Arnt1* RNA and protein have been detected in the mouse embryo adrenal with highest expression at gestation days 14–16, corresponding to adrenal development and organization [28, 29]. Mice deficient for *Ahr* did not present alterations in adrenal morphology [30] and basal plasma corticosterone levels appeared unchanged [31]. However, the corticosterone response to electroshock was impaired, indicating reduced response to stressors [31]. Mice knock-out for *Arnt1* presented reduced plasma corticosterone and absent corticosterone response to stressors in vivo and to ACTH in vitro; the adrenal gland itself, albeit without gross histological alterations, presented reduced levels of proteins associated with cholesterol transport within the cell, e.g., Star, Ldlr [32] indicating that Arnt1 is necessary for proper corticosterone production in mice. In this context, it is worth recalling that Arnt1––also known as Bmal1––is a core component of molecular circadian rhythm [33].

## Fish and marine mammals

Ichthyology has contributed significantly to the study of endocrine disruptors, with fish being the primary target of water pollution. Two main effects appear to occur: On the one side, short-term exposure to pollutants results in a stress-like cortisol increase; on the other hand, long-term exposure has been linked to decreased cortisol response to stressors, thereby possibly endangering marine wildlife survival.

### In vivo studies

Increased plasma cortisol levels after short-term incubation have been observed in several experimental models (Table 2), with agents such as phenanthrene or petroleum-derived wastewater mixtures added to tank water/feed or administered via intraperitoneal injection. Species tested range from toadfish to tilapia to trout and results appear consistent (Table 2).

**Table 2:** Experimental studies with AHR ligands on fish and marine animals in vivo and in vitro

Species	AHR ligand	Experimental model	Findings	Ref.
**In vivo studies on baseline cortisol levels**				
Pacific herring (*Clupea pallasii*)	polycyclic aromatic hydrocarbons	100 µg/l in tank for 96 h	Increased plasma cortisol	[36]
Golden gray mullet (*Chelon auratus*)	phenanathrene	0.3–2.7 µM in tank for 16 h	Increased plasma cortisol	[94]
Three-spined stickleback (*Gasterosteus aculeatus*)	mixture (offshore oil wastewater)	In tank for 72 h	Increased plasma cortisol	[95]
Gulf toadfish (*Opsanus beta*)	naphthalene phenanthrene	5 µg intraperitoneal injection, evaluation after 72 h	Increased plasma cortisol	[38]
Rainbow trout (*Oncorhynchus mykiss*)	naphthoflavone benzopyrene	10 mg/kg bw intraperitoneal injection, evaluation after 24–72 h	Increased plasma cortisol	[96]
Rainbow trout (*Oncorhynchus mykiss*)	hexachlorocyclo-hexane	0.05 mg/100 g bw intraperitoneal slow-release implant, evaluation after 18 days	Increased plasma cortisol	[97]
Freshwater tilapia (*Oreochromis* sp.)	arsenic	1–3 mg/l in tank for 20 days	Increased plasma cortisol at 96 h, reduced plasma cortisol at 20 days	[34]
Catfish (*Clarias batrachus*)	mercury chlorides	0.5 mg/l in tank for 3–6 months	Reduced plasma cortisol	[35]
**In vivo studies on cortisol response to stressor**				
Gulf toadfish (*Opsanus beta*)	naphthalene phenanthrene	5 µg intraperitoneal injection, evaluation after 72 h	Reduced cortisol response to stressor	[38]
Pacific herring (*Clupea pallasi*)	polycyclic aromatic hydrocarbons	100 µg/l in tank for 9 weeks	Reduced cortisol response to stressor	[36]
Rainbow trout (*Oncorhynchus mykiss*)	ß naphthoflavone	10 mg/kg bw in feed for 5 days	Reduced cortisol response to stressor	[39]
Rainbow trout (*Oncorhynchus mykiss*)	ß naphthoflavone	50 mg/kg bw intraperitoneal injection, evaluation after 72 h	Reduced cortisol response to stressor	[40]
Rainbow trout (*Oncorhynchus mykiss*)	ß naphthoflavone	10 mg/kg bw intraperitoneal injection, evaluation after 72 h	Increased cortisol response to acute stressor, reduced cortisol response to prolonged stress	[41]
Tilapia (*Oreochromis Mossambicus*)	polychlorinated biphenyl 126	50 µg/kg bw in feed for 5 days	Reduced cortisol response to stressor	[37]
**In vitro studies**				
Gulf toadfish (*Opsanus beta*)	- Deepwater horizon spill water - naphthalene phenanthrene	- Kidney slices from fish held for 24 h in tank containing 3% mixture	- Reduced cortisol response to ACTH	[38]
		- Kidney slices collected after 72 h 5 µg intraperitoneal injection	- Reduced cortisol response to ACTH	
Rainbow trout (*Oncorhynchus mykiss*)	ß naphthoflavone	Kidney slices incubated for 1 h at 1 µM	Reduced cortisol response to ACTH, blunted *Star* and *Cyp11a1* increase after ACTH	[39]
Rainbow trout (*Oncorhynchus mykiss*)	Endosulfan	Kidney slices incubated for 1 h at 50 µM	Reduced cortisol response to ACTH and cAMP	[42]
Tilapia (*Oreochromis Mossambicus*)	Polychlorinated biphenyl 126	Kidney slices from fish fed 50 µg/kg bw for 5 days	Reduced cortisol response to ACTH and cAMP	[37]

*bw* body weight

Biphasic results were observed during longer exposures: Increased plasma cortisol at 96 h but decreased cortisol levels at 20 days were observed in freshwater tilapia (*Oreochromis* sp.) exposed to arsenic [34]. In another freshwater species, the catfish (*Clarias batrachus*), exposed to mercury pesticides, was initially associated with increased adrenocortical activity followed by reduced plasma cortisol upon 90- and 180-day exposure [35].

Lastly, the cortisol response to stress, e.g., crowding and capture, was impaired in fish exposed to a variety of AHR ligands, from petroleum-derived hydrocarbons [36] and polychloro biphenyl congeners [37], to naphthalene, phenanthrene [38], and ß-naphthoflavone [39–41] (Table 2).

### In vitro studies

Corticosteroids are secreted by head kidney cells in fish and experiments were carried out with intrarenal tissue collected from healthy animals or fish exposed to AHR ligands in vivo. Either experimental approach resulted in reduced cortisol response to ACTH with muted increase in the rate-limiting steroidogenic enzymes, *Star* and *Cyp11a1* [39] (Table 2). Interestingly, the cortisol response to cAMP was also blunted [37, 42], indicating that impairment of cortisol release occurs downstream to the ACTH receptor. Of note, dampening of the cortisol response occurred at 20-fold lower AHR ligand concentrations compared to concentrations associated with adrenotoxic effects [42].

### Real-world studies

**Fig. 2:**
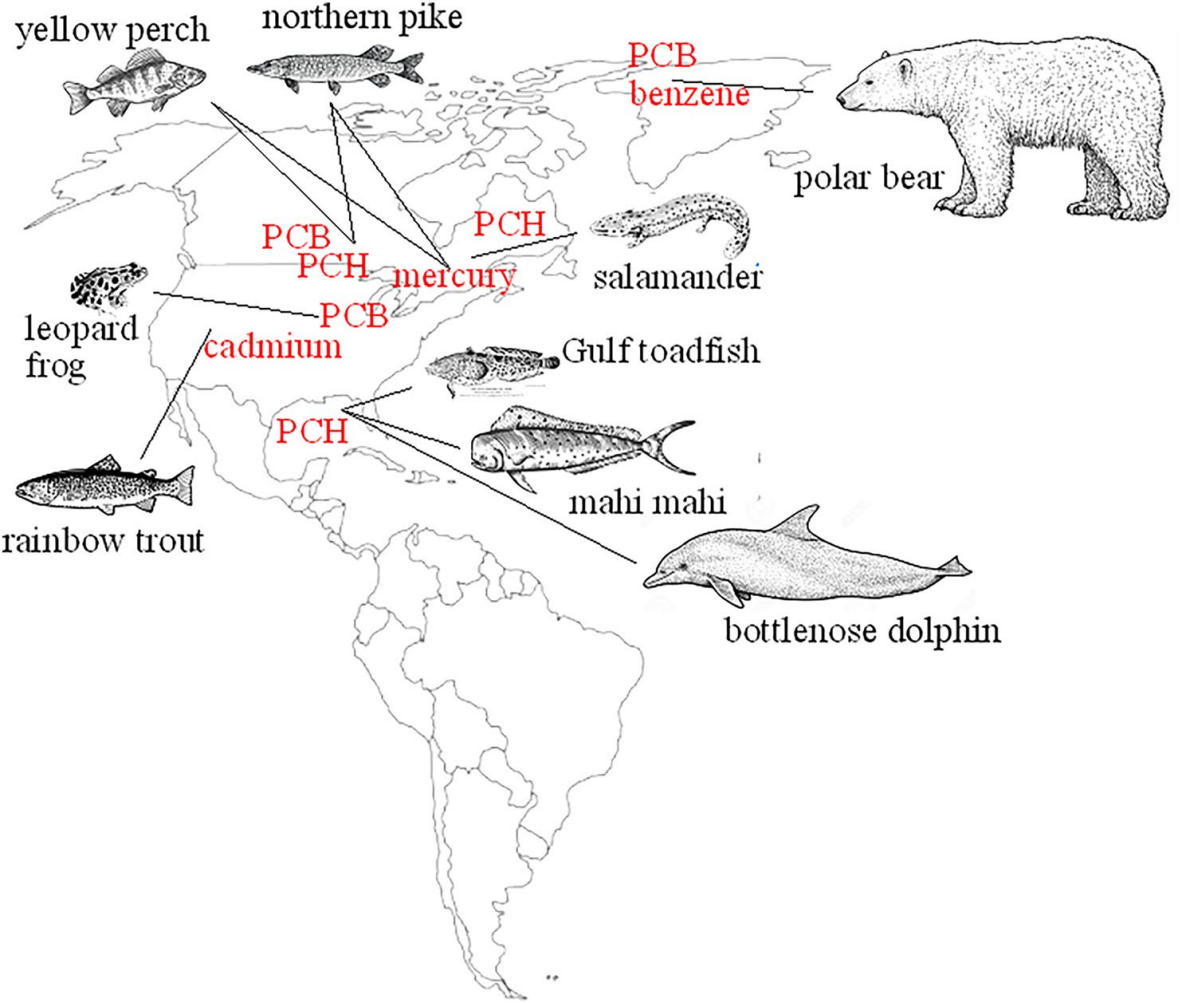
Worldwide studies on the effects of AHR ligands on cortisol secretion in free-ranging animals. *PCH* polychlorinated hydrocarbons, *PCB* polychlorinated biphenyls

In settings closer to real life (Fig. 2), fish such as yellow perch (*Perca favescens*) and northern pike (*Esox lucius*) captured from sites with high concentrations of polycyclic hydrocarbons, polychlorinated biphenyls or from bleached kraft mill effluents––containing a mixture of chlorinated chemicals––were unable to increase cortisol in response to capture stress [43, 44]. Field studies in rainbow trout (*Salmo trutta*) after the accidental leak from the Eagle mine in Colorado revealed delayed and depressed cortisol response to cage stress [45]. *Perca favescens* and *Esox lucius* resident in waters polluted with mercury from industrial drainage in the Saint Lawrence river in Canada also present an impaired cortisol response to capture stress or ACTH challenge [43, 46]. Reduced cortisol secretion was associated with the length of exposure to contaminants, as young yellow perch from smelter-contaminated lakes in Northern Canada presented normal cortisol levels, whereas, in older fish, plasma cortisol was lower compared to fish from reference sites [47].

The evidence collected so far suggests that prolonged exposure to water pollutants compromises the cortisol reserve, and this was indeed proven by studies performed after the Deepwater Horizon oil spill in 2010. The spill led to high levels of polycyclic aromatic hydrocarbons along the Gulf of Mexico coastal water [48] and exposed marine wildlife to petroleum compounds for several years. Studies on bottlenose dolphins (*Tursiops truncatus*) revealed that cortisol levels in cetaceans close to the spill were lower compared to dolphins living along the Florida coast; in fact, concentrations were subnormal in over 40% of mammals [49]. Stranded animals presented atrophied adrenals with thin adrenal cortex and reduced cortico:medullary ratio [50]. Unusual deaths were recorded after the spill, mostly due to infectious diseases; indeed, the dolphin population declined considerably following the Deepwater Horizon oil spill [51]. Normalization of cortisol concentrations in dolphins appeared to occur over time although low plasma cortisol levels were recorded for up to 4 years in some animals [52]. The effect of polycyclic hydrocarbons from the Deepwater Horizon oil spill was also assessed in another fish species, the Gulf toadfish (*Opsanus beta*). Swimming for 1 week in water fractions from the surface oil spill resulted in impaired cortisol response to stress several days after exposure, although no change in *Star*, *Cyp11a1,* or *Cyp11b1* expression was observed [53]. The authors suggested altered cholesterol availability as a possible cause and, indeed, cholesterol levels were reduced in mahi-mahi (*Coryphaena hippurus*) larvae exposed to surface oil from the Deepwater Horizon spill [54].

## Reptiles and amphibians

Reptiles and amphibians are exposed to both earth- and water-bound contaminants and represent sentinel species for endocrine-disrupting chemicals (Fig. 2)*.* Activation of the HPA axis was observed following 12- and 22-day injections of nonyl- and octylphenol surfactants in Italian wall lizards (*Podarcis sicula*), a common reptile in Mediterranean countries [55]. The animals presented increased plasma corticotrophin-releasing hormone, ACTH, and corticosterone as well as steroidogenic cell hypertrophy. Of note, adrenal morphology was altered in lizards even 2 weeks after the last injection, suggesting lasting cell alterations. Conversely, blunted corticosterone surge after confinement stress and ACTH challenge was observed in *Necturus maculosus*, an aquatic salamander living in Canadian rivers with life-long exposure to chlorinated hydrocarbons [56]. Likewise, subchronic exposure of leopard frogs (*Rana pipiens*)––an amphibian native to American Midwest wetlands––to low doses of a polychlorinated biphenyl congener resulted in decreased whole-body corticosterone and reduced corticosterone response to ACTH stimulation [57], indicative of impaired adrenal secretory capacity after chronic exposure.

## Other mammals

The polar bear (*Ursus maritimus*), a top predator with nearly 50% body fat, is among the most highly organochloride-contaminated Arctic mammals [58]. Cross-sectional analysis of pesticide exposure in polar bears from Norwegian Svalbard Islands (Fig. 2) revealed lower cortisol responses to capture stress in animals with higher plasma concentrations of organochlorides, such as hexachlorobenzene and polychlorinated byphenyl congeners [59]. Organochloride exposure was also associated with lower plasma cortisol levels in free-living Norwegian Artic polar bears [60].

The effect of petroleum-derived polycyclic hydrocarbons has also been investigated in ranch mink (*Mustela vison*), a species living close to the marine environment, with comparable results: Exposure to ship fuel oil for 2 months led to slightly reduced resting plasma corticosteroid levels and blunted cortisol response to ACTH [61, 62].

## Humans

*AHR* as well as *ARNT* and *AHRR* mRNA has been detected in human adrenal tissues, providing the basis for direct action of AHR ligands on the adrenal [63–65]. In addition, organochloride pesticides and polychlorinated biphenyls have been detected in adrenal cortex from kidney donors [66].

### In vitro studies

Several studies have been carried out using H295R, the human androgen-secreting adrenal carcinoma-derived cell line approved for the study of endocrine disruptors on testosterone and estradiol production [67]. It also allows testing for corticosteroid secretion [68], and results with AHR ligands are summarized in Table 3.

**Table 3:** Studies with AHR ligands on cortisol secretion by the human H295R cell line

AHR ligand	experimental model	findings	Ref.
6-hydroxyflavone genistein	12.5 µM for 24–48 h	Reduced cAMP-stimulated cortisol Reduced 3BHSD, 17OHD, 21OHD, 11OHD activity	[69]
prochloraz	0.1–10 µM for 24–48 h	Reduced cortisol secretion Reduced secretion of 11deoxycortisol, 17OHP	[70]
liver extracts from contaminated fish	1000–10,000 extract dilution for 48 h	Increased cortisol release Increased *CYP11B2*, *CYP11B1,* and *MC2R* expression	[71]
polychlorinated biphenyl	10 µM for 10 days	Enhanced 17OHP to cortisol conversion Increased *CYP21A2*, *CYP11B1, CYP11B2*, *MCR2* expression	[72]
α naphthoflavone ß naphthoflavone	10 µM for 24 h	Increased cortisol secretion Increased *CYP11B1* expression	[73]

*3BHSD* 3ß hydroxydehydrogenase, *17HSD* 17 alfa hydroxylase, *21OHD* 21 hydroxylase, *11OHD* 11ß hydroxylase, *17OHP* 17 hydroxyprogesterone

Reduction of cortisol secretion and microsomial and mitochondrial steroidogenic enzymes was observed with dietary AHR ligands such as 6-hydroxyflavone or genistein [69]. Likewise, steroid profiling after exposure to prochloraz revealed a dose-dependent reduction in 17 hydroxylase, 21 hydroxylase, and 3ß-hydroxydehydrogenase activity, resulting in decreased cortisol and 11-deoxycortisol [70].

Conversely, exposure of H295R cells to liver extracts from fish living in contaminated lakes resulted in marked cortisol release and increased steroidogenic enzyme expression [71]. Hexachlorobenzene and polychlorinated biphenyls were among organic pollutants in liver extracts. Similar results were observed with the polychlorinated byphenyl congener PCB126 [72] or with alfa and ß-naphthoflavone [73].

These studies also reported induction of *CYP21A2*, *CYP11B1, CYP11B2,* and *MCR2* expression [71, 72] although, interestingly, no AHR-binding elements have been detected on promoters for these genes [73, 74]. The fact that increased gene expression was observed at high doses of the compound suggests a “ripple effect” of AHR activation, rather than direct induction of steroidogenic enzyme synthesis [72]. In a similar fashion, incubation with ß-naphthoflavone revealed opposite effects on *STAR* promoter activity, with submicromolar concentrations proving stimulatory effect and higher concentrations proving inhibitory effect [63]. AHR required ARNT for maximal stimulatory activity on *STAR* and appeared to act at the SP1-binding site in the human *STAR* promoter; indeed, as with adrenal CYP genes, no consensus XRE could be identified upstream to the human *STAR* gene [63].

### Real-world setting

There are varied data on the effect of AHR ligands on cortisol secretion in human beings, mostly garnered from environmental studies. Organophosphate exposure in Thai farm workers was not associated with either reduced or increased plasma cortisol concentrations [75]; likewise, urinary cortisol metabolites measured in electrical maintenance staff exposed to polychlorobiphenyl mixtures were comparable to controls [76]. Further, plasma cortisol levels did not differ in Chinese children exposed to polychlorinated biphenyls and dioxins from electronic waste compared to children native to other regions [77].

Differing effects were recorded in women exposed during pregnancy. In farm-dwelling women from Argentina, exposure to organophosphate pesticides during pregnancy led to a reduction in plasma cortisol compared to non-exposed mothers, with a proportion of women presenting subnormal values [78]. In community-dwelling mothers from Canada, bisphenol A was detected in urine during pregnancy and associated with reduced salivary cortisol at awakening during the second trimester [79]. Conversely, in Vietnamese mothers from areas sprayed with pesticides during the Vietnam War, dioxin was detectable in breastmilk and morning cortisol in both serum and saliva was higher compared to mothers from non-exposed areas [80].

Effect on offspring of exposed mothers again proved sex dependent. Bisphenol A exposure in Canadian mothers led to newborn girls presenting higher baseline cortisol levels but lesser response to stress and the converse occurred in boys [81]. Similar results were observed in Chinese mothers exposed to phthalates, with urinary metabolite levels in mothers associated with increased cord blood cortisol in female infants and reduced cortisol levels in male infants [82].

Lastly, decreased cortisol levels were observed in individuals exposed to hydrocarbons during cleanup of the oil tanker *Prestige* spill in 2002 [83]. Reduced cortisol concentrations were most evident in young males who worked as high-pressure cleaners, as mean levels were nearly half those observed in unexposed individuals. Evaluation of cortisol plasma concentrations in fishermen 7 years after cleanup of the *Prestige* spill revealed cortisol concentrations within the normal range [84], suggesting full recovery of adrenal function.

## Other interactions between AHR and corticosteroids

Our review focused on the effect of AHR ligands on corticosteroid synthesis, but other important interactions between the two pathways are worth recalling. On the one side, several AHR ligands have been shown to interact with the glucocorticoid receptor itself. In fact, modeling of molecular docking has provided support for binding of dioxin and bisphenol A to the glucocorticoid receptor [85, 86]. To what extent endocrine disruptors mimic or antagonize glucocorticoid receptor action has yet to be fully clarified. Further, phthalates may compete with cortisol for binding to corticosteroid-binding globulin [87], thereby disrupting equilibrium between bound and available cortisol.

On the other hand, hydrocortisone has been shown to bind AHR [88] and increase* AHR* expression [89]. Indeed, integrity of the adrenal is required for full potential of AHR liver activity, as shown by studies on adrenalectomized rats [90] and in liver cells themselves [91].

## Discussion and conclusions

The adrenal gland is particularly vulnerable to endocrine-disrupting chemicals by virtue of its lipophilicity and high CYP enzyme content. At the same time, the AHR pathway stands at the crossroads of both cellular detoxification and implementation of toxic effects. Thus, the impact of AHR ligands on the adrenal carries considerable interest.

Altogether, in vitro and in vivo evidence suggests that AHR ligands exert multiple, possibly superimposed, effects on adrenal steroidogenesis. AHR ligands appear to induce an initial stress-like response followed by decreased cortisol responsivity to stimuli. Of note, AHR itself does not interact directly with steroidogenic enzyme genes, given the absence of clearly identifiable XRE-binding sites, thus additional factors are likely involved. The impairment of adrenal function may lead to altered metabolism, immune function, growth, reproduction, cardiovascular homeostasis, and, ultimately, survival. Indeed, increased mortality due to infectious diseases in dolphins with reduced cortisol secretion after the Deepwater Horizon spill provides real-world outcomes for this hypothesis.

Further studies on the impact of AHR ligands on adrenal function are of considerable clinical relevance and avenues of research could be tailored to currently available evidence. Studies carried out so far revealed that the effects of AHR ligands on adrenal secretion are determined by timing, length and degree of exposure. However, one major issue in the study of endocrine disruptors in real life is the exposure to multiple contaminants at the same time, the so-called “cocktail effect”, which does not allow clear cause–effect conclusions to be drawn. As the AHR pathway is activated by hundreds of endogenous and exogenous compounds and interacts with multiple molecular pathways, effects may prove extremely difficult to unravel. An additional layer of complexity is the bidirectional cross talk between AHR and the corticosteroid pathway, with cortisol proving essential to AHR detoxifying activity. The sex hormone milieu also plays a role, as shown by sexually dimorphic consequences of intrauterine exposure to AHR ligands in both animals and humans.

Results from exposure to individual disruptors in controlled experimental settings, e.g., rodent studies, H295R cells, are essential to establish specific features of the compound but must be placed in context with wider scoping studies. In this context, although the H295R cell line is approved for studies of endocrine disruptors [67], the limitations inherent to extrapolating findings from adrenal carcinoma to normal adrenal physiology should always be kept in mind. With this caveat, two AHR ligands with known effects on the adrenal, i.e., ketoconazole and mitotane [9, 13], have been extensively studied in H295R and are the most long-standing drugs for Cushing’s syndrome and adrenal carcinoma [98]. However, the potential impact of environmental AHR ligands on treatment response, which is known to vary over time, has yet to be investigated.

Given the pervasiveness of exposure to pollutants, some limitations––such as the “cocktail effect” or background environmental hazards––are unavoidable and may be used to advantage. In fact, the study of cortisol secretion can be linked to increasing concentrations of multiple pollutants, rather than compared to non-exposed individuals. Cortisol measurements should tailored to detect cortisol hyposecretion, i.e., morning plasma levels or response to ACTH testing, or hypersecretion, e.g., 24 h urine collections and midnight salivary samples [92]. In this context, non-invasive sampling, e.g., cortisol in urine or saliva, while easy to perform, is of little value for the detection of subnormal cortisol secretion. On the contrary, salivary cortisol could be used to assess disruption of cortisol circadian rhythm, an extremely interesting avenue of research given the links between AHR, ARNT1, and clock genes. Lastly, environmental impact on the AHR pathway is known to be involved in endocrine tumorigenesis, as shown for pituitary GH-secreting pituitary tumors [99], but the potential impact on the adrenal has yet to be established.

In conclusion, the evidence collected so far indicates that AHR ligands impact adrenal corticosteroid secretion. Several avenues of research should be pursued to provide a better understanding of its clinical consequences.
